# Menstrual cycle associated changes in hormone-related gene expression in oestrogen receptor positive breast cancer

**DOI:** 10.1038/s41523-019-0138-2

**Published:** 2019-11-15

**Authors:** Ben P. Haynes, Ophira Ginsburg, Qiong Gao, Elizabeth Folkerd, Maria Afentakis, Richard Buus, Le Hong Quang, Pham Thi Han, Pham Hong Khoa, Nguyen Van Dinh, Ta Van To, Mark Clemons, Chris Holcombe, Caroline Osborne, Abigail Evans, Anthony Skene, Mark Sibbering, Clare Rogers, Siobhan Laws, Lubna Noor, Ian E. Smith, Mitch Dowsett

**Affiliations:** 1 The Ralph Lauren Centre for Breast Cancer Research, Royal Marsden Hospital, Fulham Road, London, UK; 2 Perlmutter Cancer Center and the Department of Population Health, NYU Langone Health, New York, USA; 3 The Breast Cancer Now Toby Robins Research Centre, The Institute of Cancer Research, Fulham Road, London, UK; 4 Department of Breast Surgery, National Cancer Hospital, Hanoi, Vietnam; 5 Department of Pathology, National Cancer Hospital, Hanoi, Vietnam; 6 Department of Medicine, Division of Medical Oncology, The Ottawa Hospital and University of Ottawa, Ottawa, Canada; 7 Royal Liverpool University Hospital, Prescott Street, Liverpool, UK; 8 Yeovil District Hospital, Yeovil, Somerset UK; 9 Poole Hospital, Longfleet Road, Poole, Dorset UK; 10 Royal Bournemouth Hospital, Castle Lane East, Bournemouth, Dorset UK; 11 Royal Derby Hospital, Uttoxeter Road, Derby, UK; 12 Doncaster Royal Infirmary, Armthorpe Road, Doncaster, South Yorkshire UK; 13 Royal Hampshire County Hospital, Winchester Hampshire, UK; 14 University Hospital North Tees, Hardwick Road, Stockton-on-Tees, UK; 15 The Breast Unit, Department of Medicine, Royal Marsden Hospital, Fulham Road, London, UK

**Keywords:** Breast cancer, Tumour biomarkers, Cancer genomics

## Abstract

The major changes in hormone levels that occur through the menstrual cycle have been postulated to affect the expression of hormone-regulated and proliferation-associated genes (PAGs) in premenopausal ER+ breast cancer. Whilst previous studies have demonstrated differences in gene expression, here, we investigated if there are within patient changes in the expression of oestrogen- and progesterone-regulated genes (ERGs and PRGs) and PAGs in ER+ breast cancer during the menstrual cycle. Samples from 96 patients in two independent prospective studies of the effect of menstrual cycle on ER+ breast cancer were used. Plasma hormone measurements were used to assign tumours to one of three pre-defined menstrual cycle windows: W1 (days 27–35 and 1–6; low oestradiol and low progesterone), W2 (days 7–16; high oestradiol and low progesterone) and W3 (days 17–26; intermediate oestradiol and high progesterone). RNA expression of 50 genes, including 27 ERGs, 11 putative PRGs and seven PAGs was measured. The AvERG (geomean of *PGR*, *GREB1*, *TFF1* and *PDZK1*) was used as a composite measure of ERG expression and showed significant changes between the three windows of the menstrual cycle increasing over 2.2-fold between W1 and W2 and decreasing between W2 and W3 and between W3 and W1. Proliferation gene expression also varied significantly, following the same pattern of changes as ERG expression, but the changes were of lower magnitude (1.4-fold increase between W1 and W2). Significant changes in the expression of eight individual ERGs, including *GREB1*, *PGR* and *TFF1*, and two PAGs were observed between W1 and either W2 or W3 with all genes showing higher levels in W2 or W3 (1.3–2.4-fold; FDR 0.016–0.05). The AvProg, a composite measure of PRG expression, increased significantly (1.5-fold) in W3 compared to W1 or W2 but no significant changes were observed for individual PRGs. In conclusion, we observed significant changes in ERG, PRG and PAG expression in ER+ breast tumours during the menstrual cycle that may affect the assessment and interpretation of prominent biomarkers (e.g. PgR) and commonly used multigene prognostic signatures in premenopausal ER+ breast cancer.

## Introduction

In premenopausal women, oestrogen receptor positive (ER+) disease constitutes over 80% of breast cancers.^1^ The vast majority of these patients will receive endocrine therapy, often by ovarian ablation, although ER status is an imperfect predictor of response to this therapy. In the postmenopausal setting, short-term withdrawal of oestrogens using an aromatase inhibitor for two weeks between a woman’s diagnosis and surgery has been used to assess the oestrogen dependence of ER+ breast cancers.^2–5^ However, it is not feasible to conduct a similar pharmacological pre-surgical test in premenopausal patients as the drugs used, tamoxifen or gonadotrophin releasing hormone agonists, often display early biochemical “flare” which would obscure molecular changes over the first few weeks.^6,7^ As an alternative way of measuring a tumour’s oestrogen-dependency in the premenopausal setting, we hypothesised that if there are predictable and consistent differences in the expression of oestrogen-regulated genes (ERGs) and proliferation-associated genes (PAGs) in hormone-responsive ER+ breast tumours in premenopausal women due to the large variations in plasma concentrations of oestrogen (c.100pM to c.1000pM) and progesterone (<3 nM to >50 nM) during the menstrual cycle, the absence of such change could signify hormone insensitivity. This was driven by the observation that the expression of ERGs in ER+ breast tumours correlated strongly with circulating levels of oestradiol in postmenopausal women.^8^

In a retrospective study we found ERG expression to be 50–200% higher in mid- to late cycle (days 7–26), when oestrogen levels are higher, compared to very late or earlier in the cycle (days 27–35 and 1–6) when oestrogen levels are lower.^9^ PAGs appeared to have a lower level of expression in the progesterone-dominated luteal phase of the cycle.^10^ We also demonstrated that *RANKL* (*TNFSF11*), an archetypal progesterone-regulated gene (PRG), increased over twofold in the luteal phase.

In addition to postulating that these differences in gene expression in ER+ breast cancer might have the potential to be used in an endogenous test of endocrine responsiveness we reasoned that hormone-dependent changes in gene expression through the cycle could impact on the interpretation of commonly measured biomarkers (e.g. PgR) and multigene prognostic signatures (such as Oncotype DX Recurrence Score (RS), Prosigna (PAM50), EndoPredict (EP) and Breast Cancer Index (BCI)).^11–14^ These signatures contain multiple ERGs and PAGs, whose expression might vary in ER+ breast cancer according to when during the menstrual cycle they are measured.

The earlier studies were retrospective in nature and limited to cross-sectional comparisons between single samples from patients in pre-defined windows of the menstrual cycle. As such, while differences in tumoural gene expression through the cycle were recorded, actual changes within patients could only be implied and the variability between patients in any such changes could not be assessed. Here we directly ask (i) whether the differences noted previously are explained by consistent within patient changes in gene expression through the phases of the menstrual cycle and (ii) if these changes reflect the sensitivity of the tumours to oestrogen-deprivation and therefore can predict the antiproliferative response to ovarian ablation.

## Results

### Patient demographics

Patient demographics are described separately for the two independent prosepective studies in Supplementary Table 1. All patients were ER+, 88% were PgR+ve and 9% HER2+ve; five of the six HER2+ve cases were in the Vietnamese cohort.

### Serum hormone levels

**Fig. 1:**
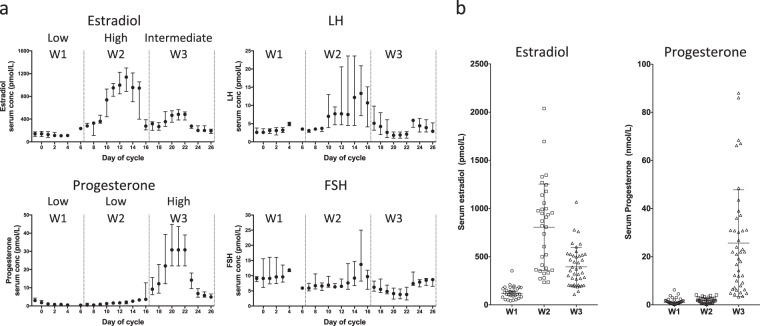
Serum hormone levels in 65 patients assessable in pre-specified window comparisons, **a** smoothed median (±1 day) concentrations, error bars indicate interquartile range, **b** comparison of oestradiol and progesterone concentrations between windows

Serum hormone concentrations showed the expected patterns of change during the menstrual cycle (Fig. 1a). Levels of oestradiol were highest in W2 (median 845 pmol/L; 95% CI 413–991) with little overlap of values in W1 (127 pmol/L; 95% CI 96–166); concentrations in W3 (364 pmol/L; 95% CI 277–468) were intermediate between those in W1 and W2 (Fig. 1b). There was nearly complete separation of progesterone concentrations in W3 (21.5 nmol/L; 95% CI 12.1–30.1) from the low values in W1 (1.1 pmol/L; 95% CI 0.7–1.7) and W2 (1.7 pmol/L; 95% CI 0.8–2.3).

### Sample availability

RNA was successfully extracted in paired tumour samples (diagnosis and surgery) from 54 of the 70 patients recruited in MenCER (Consort diagram, Supplementary Fig. 1). Nine of these patients were excluded due to ambiguous menstrual cycle data. Overall, 45 patients with good quality gene expression and consistent menstrual cycle data were available. The median time between the tumour samples was 27 days (interquartile range 20–34 days).

In the Vietnamese study, paired FFPE tumour samples at diagnosis and two weeks later (prior to OvX) were available for 35 of 56 patients (Consort diagram, Supplementary Fig. 1). Of these, RNA was successfully extracted for 27 sample pairs, seven of which were excluded due to ambiguous menstrual cycle data, yielding a final set of 20 patients. The median time between the tumour samples was 9 days (interquartile range 8–13 days).

Combining samples from the two studies gave a final group of 65 patients with the following window comparisons available: 15 same window comparisons (all MenCER); 14 comparisons of W1 vs. W2 (7 MenCER, 7 Vietnamese); 20 comparisons of W2 vs. W3 (14 M, 6 V); 10 comparisons of W1 vs. W3 (7 M, 3 V); 28 comparisons of W1 vs. (W2 or W3) (14 M, 14 V); 32 comparisons of (W1 or W2) vs. W3 (23 M, 9 V) (Supplementary Fig. 2, Supplementary Data 1).

### Unsupervised clustering of combined sample set

**Fig. 2:**
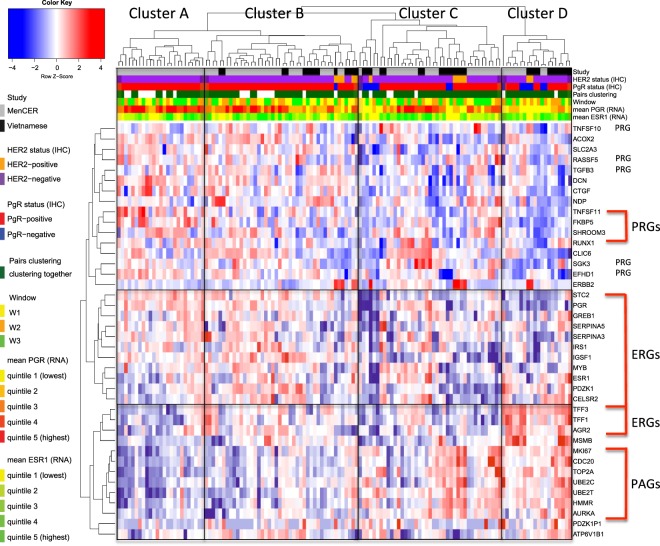
Unsupervised hierarchical clustering of gene expression data from the combined sample set of 130 samples (65 pairs)

Unsupervised clustering of the gene expression data from this combined sample set of 130 samples showed that 52 of the 65 paired samples clustered together. There were four main clusters apparent, labelled A–D in Fig. 2. Cluster A was enriched for samples taken in W3 (24% W1, 24% W2, 52% W3) and showed the lowest relative expression of PAGs, high expression of most ERGs and high PRG expression. Cluster B comprised a more evenly mixed group of tumours from the three windows (36% W1, 32% W2, 32% W3) and segregated from cluster A based on relatively higher PAG and lower PRG expression. Clusters C and D differed from the other clusters on the basis of their lower expression of ERGs and PRGs and contained 94% (15/16) of the PgR -ve samples. Cluster D was enriched for tumour samples taken in W2 (25% W1, 45% W2, 30% W3) and showed the highest relative expression of PAGs and the lowest of PRGs and *PGR* (30% IHC PgR −ve). Overall, in this combined dataset, *PGR* expression and the AvProg were both inversely correlated to the expression of PAGs (Spearman *r* = −0.29, *p* = 0.0007 and *r* = −0.36, *p* = 0.0007, respectively) (Supplementary Fig. 3).

### Comparison of gene expression changes in samples from the same window of the menstrual cycle

We first assessed if there were any significant changes in gene expression between the 15 tumour samples taken in the same window of the menstrual cycle (but one cycle apart). Five of our 45 selected genes (*GEM*, *NFKB1A*, *PTGS2*, *STAT5A*, *ZFP36*) increased significantly (1.6–4.4-fold; FDR 0.04 to 0.0025; Supplementary Data 2, Supplementary Fig. 4) between diagnosis and surgery. It appears that the upregulation of these genes may be related to longer time to fixation for the surgical samples rather than study-related as four of these genes were also up regulated in our studies of changes in gene expression in the absence of drug-treatment.^15,16^ These five genes were excluded from further analysis, as any changes in their expression between windows could not be ascribed with confidence to menstrual cycle effects.

### Gene expression changes during the menstrual cycle: individual window comparisons

For the individual window comparison between W1 and W3 and between W1 and W2, the sample size was small (*n* = 10 and 14 pairs respectively) and none of the changes in individual gene expression reached significance after correction for multiple testing (FDR < 0.05; Supplementary Data 2). However, for the comparison between W1 and W2 whilst no gene had an FDR < 0.05, the four ERGs comprising the AvERG increased 1.9–2.7 fold in W2 compared to W1 (uncorrected *p*-values 0.003 to 0.068, FDR 0.068 to 0.23). The expression of all seven PAGs also showed a consistent trend to increase between W1 and W2 (FC 1.1–1.5; uncorrected *p*-values 0.002 to 0.19, FDR 0.13 to 0.36; Supplementary Data 2).

Between W2 and W3 (*n* = 20 pairs), *TFF1* and *ATP6V1B1* both decreased significantly (FC 0.47, BH = 0.008; FC 0.55, BH = 0.034, respectively; Supplementary Data 2). Of note *FKBP5*, a putative PRG, increased in 16 of the 20 tumours and this neared statistical significance (FC 1.6, BH = 0.051). *RANKL*, which was expressed to a very low extent, and other putative PRGs (*RASSF5*, *EFHD1*, *TGFB3*, *SGK3*, *SHROOM3*, *TNFSF10*) did not consistently change between W2 and W3.

**Fig. 3:**
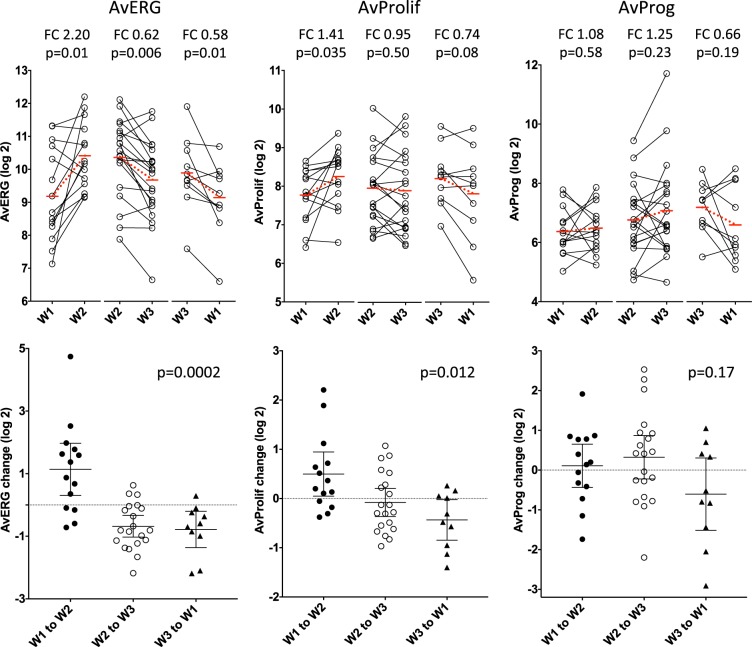
Changes in AvERG, AvProlif and AvProg between individual windows (FC; fold-change). Dotted red lines indicate change in mean level between compared windows. Error bars indicate mean ± 95% CI

The AvERG showed significant changes between the three windows (Kruskal-Wallis *p* = 0.0002); increasing between W1 and W2 (FC 2.2, *p* = 0.011), and decreasing between W2 and W3 (FC 0.62, *p* = 0.006) and between W3 and W1 (FC 0.58, *p* = 0.01) (Fig. 3). Proliferation gene expression (AvProlif), also varied significantly (Kruskal-Wallis *p* = 0.012), increasing between W1 and W2 (FC 1.41, *p* = 0.035) with non-significant decreases thereafter between W2 and W3 (FC 0.95, *p* = 0.50) and W3 to W1 (FC 0.74, *p* = 0.084). The AvProg showed a numerically higher level in W3 compared to the other windows but this did not approach statistical significance (FC 1.2–1.6, *p* = 0.19–0.23; Fig. 3).

### Gene expression changes during the menstrual cycle: Window 1 vs. Window 2 or 3

A pre-specified comparison of W1 vs. a combined W2 or W3 (*n* = 28) allowed for greater power for assessing the change from a low to high/intermediate oestradiol exposure. The expression of 10 individual genes (eight ERGs and two PAGs) changed significantly between W1 and W2 or W3, with all genes showing higher levels in W2-W3 (FC 1.4–2.4; FDR 0.016–0.05; Supplementary Table 2). These genes included three of the four archetypal ERGs (*GREB1*, *PGR* and *TFF1*) comprising the AvERG and five putative ERGs (*IGSF1*, *MSMB*, *SERPINA3*, *ATP6V1B1*, *CELSR2*) whose expression was previously shown to be down-regulated after OvX.^17^ *IGSF1* and *MSMB* showed the greatest magnitude of change between the windows (FC 2.4 and 2.3-fold respectively). In agreement with the gene expression data, mean protein levels of PgR increased between W1 and W2 or W3 (18.3% increase, *p* = 0.0015, FDR 0.024; Supplementary Fig. 5) but this did not lead to a change in PgR positive/negative status for any tumour. Protein levels of ER and Ki67 did not show a statistically significant change between W1 and W2 or W3 (*p* = 0.056, BH 0.11 and *p* = 0.33, BH = 0.42 respectively; Supplementary Fig. 5).

**Fig. 4:**
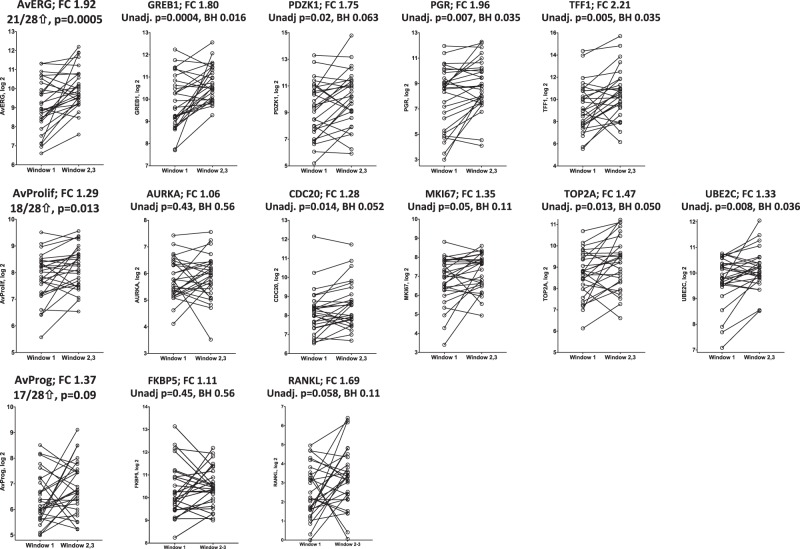
Changes in AvERG, AvProlif and AvProg and in expression of their individual component ERGs, PAGs and PRGs between Window 1 vs. Windows 2 or 3 (*n* = 28)

The AvERG (FC 1.9, *p* = 0.0005) and AvProlif (FC 1.3, *p* = 0.013) increased significantly between W1 and W2-W3 (Fig. 4). The AvProg response was more variable but there was a trend to increase in W2-W3 (FC 1.4, *p* = 0.09). The changes in AvERG and AvProlif showed a weak correlation but there was no obvious relationship between AvProg and either AvERG or AvProlif (Supplementary Fig. 6). Individually the genes comprising the AvERG increased approximately twofold in W2-W3 compared to W1 (FC 1.7–2.2; BH 0.011–0.06) (Fig. 4). A similar pattern was observed for PAGs with the exception of *AURKA*, although the change was not as large (FC 1.3–1.5, BH 0.035–0.11) (Fig. 4).

### Correlation of response to oestrogen deprivation with the change in AvERG between W1 vs. W2-W3

**Fig. 5:**
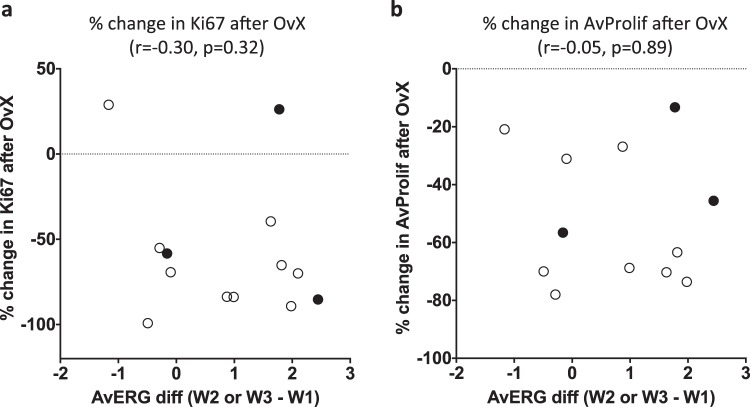
Correlation of change in AvERG during menstrual cycle (Window 1 vs. Windows 2 or 3) with % change in Ki67 (**a**) and % change in AvProlif (**b**) after OvX; • HER2+ ve tumours

There was no correlation of the response to OvX in the Vietnamese study, as measured by % change in Ki67 (*r* = −0.30, *p* = 0.32) or AvProlif (*r* = −0.05, *p* = 0.89), with the change in AvERG between W1 vs. W2–W3 in the small group of patients in which this could be compared (*n* = 12–13) (Fig. 5). Anecdotally, there was only one clear HER2-ve non-responder to OvX by Ki67 and this showed the least increase in AvERG between W1 vs. W2-W3.

### Gene expression changes during the menstrual cycle: Window 3 vs. Window 1 or 2

The effects of the higher level of progesterone in W3 (roughly corresponding to the luteal phase) compared to the rest of the cycle were examined by comparing gene expression changes between W3 vs. a combined W1 or W2 (*n* = 32) (Supplementary Table 3, Supplementary Fig. 7). For this comparison, *FKBP5*, a putative PRG, was the most significant gene and showed the greatest fold-change (FC 1.5, *p* = 0.0057, BH = 0.23), increasing in 24 of 32 tumours in W3. *RANKL* was the 5th most significant gene and showed the second greatest increase (FC 1.4, *p* = 0.10, BH = 0.65). Other putative PRGs changed to a lesser extent and with less significance. Overall, AvProg increased significantly in W3 (FC 1.5, *p* = 0.026) but there were no significant changes in AvERG or AvProlif. The change in AvProg showed a borderline significant correlation with PGR expression (*r* = 0.34, *p* = 0.06), such that the tumours with higher PGR expression showed a greater change in AvProg.

### Unpaired analysis of differences in gene expression through menstrual cycle

**Fig. 6:**
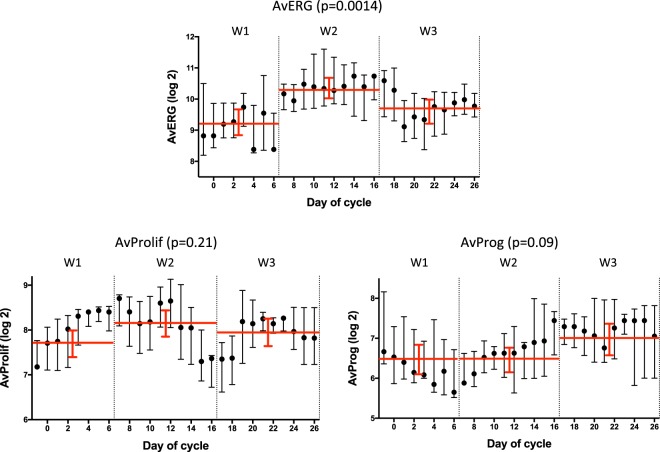
Differences in AvERG, AvProlif and AvProg during the menstrual cycle (*n* = 103, unpaired); smoothed median (±1 day) values, error bars indicate interquartile range, red lines show mean (±95% confidence interval) of each window

To determine the consistency of these data with our earlier cross-sectional analysis we performed an exploratory unpaired analysis of all samples assessing the differences in gene expression between the three pre-defined windows (103 samples; 29 in W1, 37 in W2, 37 in W3). The AvERG showed significant differences between the three windows (KW *p* = 0.0014) with the highest level in W2; W1 vs. W2 (FC 2.2; *p* = 0.0005), W2 vs. W3 (FC 0.63, *p* = 0.015), W1 vs. W3 (FC 0.73, *p* = 0.14) (Fig. 6). The AvProlif showed a trend to increase between W1 and W2 (FC 1.3, *p* = 0.07) and there was a strong trend for the AvProg to be higher in W3; W2 vs. W3 (FC 1.5, *p* = 0.056), W1 vs W3 (FC 0.66, *p* = 0.07). Hierarchical clustering of the unpaired gene expression data according to the window in which the sample was taken further illustrates the differences in gene expression between windows and also demonstrates the large variation that occurs within each window (Supplementary Fig. 8).

## Discussion

We have previously reported significant differences in the expression of ERGs, PAGs and the PRG, *RANKL*, in ER+ tumours in a retrospective study that related to the major changes in hormone levels that occur during the menstrual cycle.^9,10^ Here, we have extended that observation in a prospective study to show significant changes of gene expression of ERGs, PRGs and PAGs within individual patients through the menstrual cycle and the degree of variability in these changes between patients.

Previous work investigating changes in breast tumour biology during the menstrual cycle is limited and the data are very variable^18–25^ which is most likely due to difficulties and differences in assigning the phase of menstrual cycle. Here, both serum hormone concentrations and menstural cycle data were used to improve the definition of times through the mentrual cycle and to assign patients to one of three pre-defined menstrual cycle windows. Approximately 20% of cases were excluded due to inconsistent menstrual cycle data. Windows 1 and 2 had low and high E2 levels, respectively (up to a sevenfold difference) and very low progesterone levels; window 3, which largely represents the luteal phase, had mean levels of E2 intermediate between those of windows 1 and 2 and up to 20-fold higher levels of progesterone.

We combined tumour samples from patients in two independent trials to increase the power of the study as individually the separate studies would have been too small to reach meaningful conclusions. Whilst the trials had some differences in their design and patient demographics, unsupervised clustering of the gene expression data generated did not reveal any clear study-related effects.

Tumour samples taken in the same window of the menstrual cycle provided a control group and indicated that the expression of five of our selected genes increased between diagnosis and surgery irrespective of cycle window. The upregulation of these genes does not appear to be study-related as four of these genes were also upregulated in studies of changes in gene expression in the absence of drug-treatment^15,16^ where the upregulation was ascribed to differences in the time taken to fixation for the samples at the two time points. These five genes were thus excluded from further analysis, as any changes in their expression between different windows would not be attributable to menstrual cycle effects.

Unsupervised clustering of the gene expression data from all samples showed that most pairs clustered together indicating that the changes in gene expression during the menstrual cycle were not as great as the variation between patients in the majority of cases. Nonetheless, the clustering revealed contrasting clusters enriched for tumours taken in W2 and W3 indicating the timing of the menstrual cycle had some effect on the clustering. The cluster enriched for W2 tumours showed the highest relative expression of PAGs and the lowest of PRGs while the cluster enriched for W3 tumours showed the lowest relative expression of PAGs and high PRG expression. This was reflected by an inverse correlation between both *PGR* expression and signalling and proliferation across all samples. These data are consistent with progesterone receptor signalling modulating oestrogen-driven proliferation in this premenopausal setting. The concept that PR activation in the context of oestrogen-driven, ER+ breast cancer, can have an anti-proliferative effect has been postulated by others^26,27^ and it seems that the oestrogenic status can directly affect whether progestogens are pro-proliferative or antiproliferative. Thus, in the absence of a functional oestrogen-activated ER complex, PgR activation can stimulate proliferation^28–30^ but when oestrogen and a progestogen are combined, reductions in the oestrogen-induced growth response have been reported both in vitro^28,31^ and ex vivo.^26^ Mechanistically, it appears that in the presence of both oestrogen and progesterone ligands, PR can affect ER target gene activity by altering the interaction between ER and chromatin thereby changing the transcriptional output of the ER complex.^26,27^

The comparison of gene expression between W1 (low oestradiol) with W2 (high oestradiol) was the most biologically straightforward window comparison in terms of hormone levels and this revealed a strong trend for an increase in ERG expression between W1 and W2. Thus, the four ERGs comprising the AvERG (a pre-defined composite measure of ERG expression) all increased two to threefold in W2 compared to W1 but this did not reach statistical significance most likely due to the small sample size available. Comparison of W2 and W3 is less straightforward to interpret as changes could be due to either the lower oestradiol levels (approximately 50%), or the much higher progesterone levels in W3 (>10-fold) or both; the only two genes that changed significantly were ERGs (*TFF1*, *ATP6V1B1*) which both decreased by approximately 50% in W3. The AvERG, showed significant changes between the three individual windows with a mean increase of 220% between W1 and W2 and approximate 40% decreases thereafter between W2 and W3 and between W3 and W1.

To increase the power to detect changes in gene expression from a low to high/intermediate oestradiol exposure we performed a pre-specified comparison of W1 vs. a combined W2 or W3. This showed that the expression of eight ERGs significantly increased (mean of 1.9-fold) between W1 and W2 or W3 including three of the four ERGs (*GREB1*, *PGR* and *TFF1*) comprising the AvERG. Similar to our earlier retrospective study,^9,10^ the fourth gene comprising the AvERG, *PDZK1*, did not show a significant change in expression although it showed a strong trend to do so. This is in agreement with a recent study which showed a correlation of *GREB1*, *PGR* and *TFF1* but not *PDZK1* gene expression in ER +ve tumours with serum oestradiol levels in premenopausal patients.^32^ This latter study concurred with our earlier cross-sectional study but lacked the longitudinal aspect of the current study to allow consideration of within patient changes. Overall, the AvERG showed a near twofold increase in expression between W1 and W2 or W3. This compared to a difference of 1.5-fold between the same windows in the retrospective study.^9,10^ Of the other putative ERGs that changed significantly between W1 and W2 or W3, *IGSF1*, a member of the immunoglobulin-like domain-containing superfamily and *MSMB*, an immunoglobulin binding factor showed the greatest magnitude of change. Both of these genes have been reported to have inhibin activity,^33,34^ which may be a factor in their increase in expression in W2–W3 vs. W1.

An unpaired analysis to assess differences rather than changes in gene expression between the three pre-defined windows demonstrated that the AvERG showed significant differences between all windows, mirroring the paired changes reported above, and these showed the same pattern to the previous cross-sectional retrospective study.^9,10^

The expression of PAGs across the menstrual cycle mirrored the changes observed in ERG expression but the magnitude of effect was less. Thus, between W1 and W2, individual PAGs showed a trend to increase between W1 and W2 such that the AvProlif, a pre-defined composite measure of PAG expression, showed significant changes between the three windows characterised particularly by a mean increase of 40% between W1 and W2. Comparison of W1 vs. W2 or W3 revealed significant increases in the expression of two individual PAGs and a 30% increase of the AvProlif in W2 or W3 compared to W1. These data are in slight contrast to our earlier report of lower PAG expression in W3 with no difference between W1 and W2.^9,10^ However, the unpaired analysis of the current data demonstrated a very similar pattern of expression of AvProlif to the previous retrospective study with a decline in proliferation at the end of W2 (days 13–16) such that the AvProlif is near its nadir at the start of W3.

The RANK signalling pathway is known to be a key paracrine mediator of progestogen action in breast epithelium and its protein expression has been shown to correlate with serum progesterone levels.^35,36^ We previously reported twofold higher levels of *RANKL* in W3, roughly corresponding to the luteal phase of the menstrual cycle, when progesterone levels are at their highest, compared to the other windows^10^ and this has also been observed by others.^37^ Here, we measured *RANKL* and 10 other putative PRGs to investigate if changes in their expression during the menstrual cycle were apparent and used the AvProg as a composite measure of PRG gene expression. Whilst the number of samples available between W1 and W3 was too small to detect any significant changes in individual gene expression, comparison of W2 and W3 showed that *FKBP5* increased in 80% of the tumours in W3 and this was borderline significant. However, *RANKL* itself did not show an increased level of expression in W3, possibly because it was expressed to a very low extent in this group of tumours. The AvProg did not show any significant changes between the individual windows but showed a trend to increase in W3 compared to the other windows. A comparison of W3 with a combined W1 or W2 increased the power to compare the effects of a high progesterone milieu in W3 with the much lower progesterone levels in W1 or W2 and demonstrated that the AvProg increased significantly in W3 as would be expected due to the much higher level of progesterone in W3 compared to the rest of the cycle. These changes in PRGs were more modest than those in ERG and PAG expression across the cycle as a whole.

In order to investigate if the changes in gene expression in ER+ breast cancer during the menstrual cycle we report here could predict the antiproliferative response to ovarian ablation we correlated the change in AvERG between W1 and W2 or W3 with the response to OvX in samples from the Vietnamese study.^17^ Only a very weak correlation was observed. Although this analysis had only low power due to the small number of samples available, even with this small number it appears that this putative endogenous test of hormonal sensitivity could not be sufficiently reliable for clinical use.

The main weakness of the current work was the relatively low proportion of patients available for comparisons of the individual windows of the menstrual cycle, even after combining patient samples from two independent studies. Combining windows enabled greater power but in some cases (e.g. combining W2 and W3) also added possible confounding factors such as widely differing progesterone concentrations within the combined window. Despite this, the impact of changes in endogenous reproductive hormones on gene expression was demonstrated very clearly.

The multigene prognostic signatures which are commonly used in ER+ breast cancer such as RS, PAM50, EP and BCI,^11–14^ contain mulitple ERGs and PAGs and it is well established that the expression of these genes are two of the main drivers of the scores obtained from these tests.^38^ Thus, the possibility that the hormonal changes during the menstrual cycle could affect the read out from these signatures merits study and we are investigating this hypothesis in a follow-up study.

In conclusion, our data indicate that there are significant changes in ERG, PRG and PAG expression in line with the hormone changes that occur during the menstrual cycle. These changes need to be noted in any studies of the hormone-related biology of breast cancer in premenopausal patients and may affect the interpretation of data from some of the multigene prognostic signatures which are commonly used in ER+ breast cancer.

## Methods

### Patients and study designs

Patients were drawn from two prospective studies that assessed the possible impact of the menstrual cycle on breast tumour biology in ER+ breast cancer. The first of these was MenCER (The Menstrual cycle as a test of endocrine responsiveness in premenopausal breast cancer), a UK-based multicentre study. In addition, menstrual cycle effects were studied in patients from a study of neoadjuvant oophorectomy (OvX) in Vietnam. The endocrine and molecular effects of OvX in this Vietnamese study have been recently published.^17^

For the MenCER study, premenopausal women aged <50 years old with histologically confirmed ER+ breast cancer who were proceeding to surgery with no pre-surgical therapy, had a regular menstrual cycle and had not received previous cancer therapy were eligible. Ethical approval for the study was received from the local research ethics committee (South West London REC 3). All participants provided written informed consent. Paired blood and tumour samples were taken both at diagnosis and at surgery with no treatment occurring between these time-points. Patient reported date of last menstrual period and length of cycle were recorded. The timing of surgery in relation to menstrual cycle was not controlled in any way and it was expected that the menstrual phase at the time of diagnosis and surgery for any given subject would be a chance event. We also reasoned that the time between diagnosis and surgery would be approximately two weeks and therefore in most patients would involve different parts of their menstrual cycle. An accrual target of 70 patients was selected on the basis of power calculations based on the magnitude of changes (twofold) in ERGs we observed in our previous retrospective studies.^9,10^ With 70 paired comparisons between the groups it would be possible to detect a standardised difference of 0.5 between the groups for each of the biomarkers with 80% power, and a standardised difference of 0.6 with 90% power.

The Vietnamese study was a single arm study of neoadjuvant OvX in patients with ER+ breast cancer, details of which have been described previously.^17^ In brief, the study recruited 56 premenopausal women with ER+ breast cancer for whom modified radical mastectomy and surgical bilateral salpingo-oophorectomy was planned as part of their breast cancer treatment. Patients had to report regular menstrual cycles and must not have received any prior chemotherapy or radiotherapy for their cancer. The study was approved by the Institutional Ethics Committee of the National Cancer Hospital, Hanoi, Vietnam from where all study participants were recruited and by the Research Ethics Board of the University of Toronto, Canada, from where the study was coordinated. The Committee for Clinical Research at the Royal Marsden Hospital, London approved the analysis of the samples collected in this trial. All participants provided written informed consent. Breast tumour core biopsies (formalin fixed and paraffin embedded) taken at diagnosis (A) and intra-operatively at the time of OvX (B; 2 weeks later) were used in the current study. No treatment occurred between these time-points. A single blood sample taken pre-OvX (on day of OvX prior to anaesthesia or pre-operative medication; timepoint B) was available for hormone measurements. Similar to the MenCER study, the timing of surgery in relation to menstrual cycle was not controlled.

Exclusion criteria for both studies included: metastatic disease, use of oral contraceptives or other hormonal contraceptives and concomitant use of medications known to influence oestrogen levels. Informed consent was obtained from all participants.

### Serum hormone measurements

Serum concentrations of oestradiol (E2) were measured by radioimmunoassay following pre-assay purification using an organic extraction as described previously.^39,40^ Progesterone was measured using a solid phase radioimmunoassay (Beckman Coulter IM1188). LH and FSH were measured using immunoradiometric assays (IBL International MG12151 and Diasource KIP0841 respectively). The serum hormone measurements were used in combination with menstrual cycle information to ascribe patients to one of three pre-defined menstrual cycle windows prior to consideration of the biomarker data: window 1 (W1; low oestrogen and progesterone milieu) days 27–35 or 1–6; window 2 (W2; high oestrogen and low progesterone milieu) days 7–16; window 3 (W3; intermediate oestrogen and high progesterone milieu) days 17–26 (Supplementary Fig. 2). In six patients one of the sample pairs fell on the cusp of two windows (two between W1 and W2, four between W2 and W3; Supplementary Data 1); these samples were not used in the individual window comparisons but were included when W1 and W2 or W2 and W3 were combined.

### Immunohistochemistry

Hematoxylin and eosin sections were prepared for all FFPE tumour samples and were reviewed to confirm diagnosis and assess tumour content. Samples with tumour content <40% were excluded from further analysis. Immunohistochemistry (IHC) and scoring for ER, PgR and Ki67 were performed as reported previously.^41,42^ ER and PgR were considered positive if ≥1% cells stained positive. HER2 was measured immunohistochemically using the HercepTest (DakoCytomation) and by fluorescent in situ hybridization (Vysis Pathvysion, Downers Grove, IL) according to manufacturer’s instructions. HER2 was considered positive if immunohistochemical staining was scored 3+ or 2+ if the fluorescence in situ hybridization analysis indicated an amplification ratio of >2.0.^41^

### Gene selection

Selection of genes for analysis in the biopsies was based on the set of genes we measured previously in a retrospective cross-sectional study.^10^ This previous set of genes comprised *ESR1*, 26 ERGs and putative ERGs that correlated with variations in plasma E2 concentrations, seven PAGs and a single PRG, *RANKL*. ERGs whose expression did not differ in the retrospective study were replaced with 16 other ERGs and putative ERGs based on the observation that they were the most highly up or down-regulated after OvX.^17^ For the PAGs, we selected four of those measured previously plus three other PAGs that were the most down-regulated after OvX. In addition to *RANKL*, we selected 10 other putative PRGs for measurement based on commonality in at least two literature reports^26,43–48^ and Mohammed^20^ personal communication. In total, 45 genes of interest (18 genes in common with the previous study^10^) were selected for gene expression measurement (Table 1). We used the AvERG (average ERGs; the geomean of *PGR*, *GREB1*, *TFF1* and *PDZK1*^8^) as a composite measure of ERG expression, the AvProlif (average PAGs; geomean of *AURKA*, *CDC20*, *MKI67*, *TOP2A*, *UBE2C*) as a composite measure of PAGs and the AvProg (average PRGs; geomean of *FKBP5* and *RANKL*) as a composite measure of PRG expression. We also measured five housekeeping genes (*ACTB*, *MRPL19*, *PSMC4*, *SF3A1*, *TBP*).

**Table 1:** Genes selected for measurement and their hormone-dependency

	Oestrogen-regulated genes	Proliferation-associated genes	Putative progesterone-regulated genes	Differences in expression during menstrual cycle^9,10^	Down-regulated after OvX^17^	Upregulated after OvX^17^
ESR1						
ERBB2						
ACOX2	x			x	x	
AGR2	x			x	x	
ATP6V1B1	x				x	x
CELSR2	x				x	
CLIC6	x				x	x
CTGF	x					x
DCN	x					x
GEM	x					x
GREB1	x			x	x	
IGSF1	x				x	x
IRS1	x				x	x
MSMB	x				x	x
MYB	x				x	
NDP	x					x
PDZK1	x			x	x	
PDZK1P1	x				x	x
PGR	x			x	x	
PTGS2	x					x
RUNX1	x			x	x	
SERPINA3	x			x	x	
SERPINA5	x			x	x	
SLC2A3	x					x
STC2	x			x	x	
TFF1	x			x	x	
TFF3	x				x	x
TGFB3	x		x			x
ZFP36	x		x			x
EFHD1			x			
FKBP5			x		x	
NFKBIA			x			
RANKL (TNFSF11)			x	x		
RASSF5			x			
SGK3			x			
SHROOM3			x			
STAT5A			x			
TNFSF10			x		x	x
AURKA		x			x	
CDC20		x		x	x	
MKI67		x		x	x	
TOP2A		x		x	x	
HMMR		x			x	
UBE2C		x			x	x
UBE2T		x			x	x

### Measurement of gene expression

Total RNA was extracted from two 10 μm sections of formalin-fixed paraffin-embedded breast tumour samples using the RecoverAll (Ambion) kit according to the manufacturer’s procedure. The NanoString nCounter gene expression system (GEN2) (NanoString Technologies, Seattle, WA) was used to measure gene expression without target amplification.^49^ In brief, an nCounter CodeSet (NanoString Technologies) containing gene-specific probe-pairs for the 50 genes selected above (including the five housekeeping genes) as well as six exogenous positive control RNA targets, and eight exogenous negative control sequences, was hybridised to 200 ng total RNA in a single 30 μl hybridization reaction. After overnight hybridization (15–21 h) at 65 °C, the samples were processed using the NanoString nCounter Prep Station and Digital Analyzer according to the manufacturer’s instructions. Assay validity was confirmed by assessing the linearity of six internal positive RNA controls and the non-specific background from signal in eight internal negative controls included in each reaction. The raw nCounter counts for each gene of interest were corrected for background by subtracting the geometric mean of the negative controls, normalised to the geometric mean of the five housekeeping genes to allow for variation in the amount and quality of input RNA and log2 transformed (Supplementary Data 1; raw gene expression data; Supplementary Data 3; normalised log-transformed data). The housekeeping genes did not show any significant changes in expression between individual windows or between the combinations of windows (Supplementary Table 4).

### Data analysis

For paired data, we performed pre-specified analyses of changes in the gene expression levels between individual windows (W1 vs. W2, W2 vs. W3, W3 vs. W1) and combinations of windows (W1 vs. [W2 or W3]; [W1 or W2] vs. W3) using the Wilcoxon matched pairs signed rank test. For unpaired data, differences in gene expression between the three windows were compared using the Kruskal-Wallis test and differences in gene expression levels between the specific windows were assessed using the Mann Whitney test (Graphpad Prism 7). To calculate the AvERG, AvProlif and AvProg the geometric mean of the individual gene expression values of the indicated genes was taken and log-transformed. For individual genes the Benjamini–Hochberg procedure was used to the calculate false discovery rate (FDR) in order to adjust for multiple testing.

### Reporting summary

Further information on research design is available in the Nature Research Reporting Summary linked to this article.

## Supplementary information

Reporting Summary Checklist

Supplementary information

Supplementary Data 1

Supplementary Data 2

Supplementary Data 3

## Data Availability

Datasets supporting Fig. 1, Supplementary Table 1 and Supplementary Fig. 5 of this published article, are publicly available in the figshare repository, 10.6084/m9.figshare.9892211.^50^ All the other datasets supporting the findings of this study are available in the supplementary files of the published article.

## References

[1] Dodson A (2018). Breast cancer biomarkers in clinical testing: analysis of a UK national external quality assessment scheme for immunocytochemistry and in situ hybridisation database containing results from 199 300 patients. J. Pathol. Clin. Res.

[2] Smith I (2005). Neoadjuvant treatment of postmenopausal breast cancer with anastrozole, Tamoxifen, or both in combination: the Immediate Preoperative Anastrozole, Tamoxifen or Combined with Tamoxifen (IMPACT) multicentre double-blind randomized trial. J. Clin. Oncol..

[3] Dowsett M (2007). Prognostic value of Ki67 expression after short-term presurgical endocrine therapy for primary breast cancer. J. Natl. Cancer Inst..

[4] Ellis MJ (2008). Outcome prediction for oestrogen receptor-positive breast cancer based on post-neoadjuvant endocrine tumour characteristics. J. Natl. Cancer Inst..

[5] Freedman OC (2010). A randomized trail exploring the biomarker effects of neoadjuvant sequential treatment with exemestane and anastrozole in post-menopausal women with hormone receptor-positive breast cancer. Breast Cancer Res. Treat..

[6] Klijn JG (2000). Combined treatment with buserelin and tamoxifen in premenopausal metastatic breast cancer: a randomized study. J. Natl. Cancer Inst..

[7] Jannuzzo MG (2009). Oestrogen suppression in premenopausal women following 8 weeks of treatment with exemestane and triptorelin versus triptorelin alone. Breast Cancer Res Treat..

[8] Dunbier AK (2010). Relationship between plasma oestradiol levels and oestrogen-responsive gene expression in oestrogen receptor-positive breast cancer in postmenopausal women. J. Clin. Oncol..

[9] Haynes BP (2013). Expression of key ooestrogen-regulated genes differs substantially across the menstrual cycle in ooestrogen receptor-positive primary breast cancer. Breast Cancer Res. Treat..

[10] Haynes BP (2014). Differences in expression of proliferation-associated genes and RANKL across the menstrual cycle in oestrogen receptor-positive primary breast cancer. Breast Cancer Res. Treat..

[11] Paik S (2004). A multigene assay to predict recurrence of tamoxifen-treated, node-negative breast cancer. N. Engl. J. Med..

[12] Parker JS (2009). Supervised risk predictor of breast cancer based on intrinsic subtypes. J. Clin. Oncol..

[13] Filipits M (2011). A new molecular predictor of distant recurrence in ER-positive, HER2-negative breast cancer adds independent information to conventional clinical risk factors. Clin. Cancer Res..

[14] Jerevall PL (2011). Prognostic utility of HOXB13:IL17BR and molecular grade index in early-stage breast cancer patients from the Stockholm trial. Br. J. Cancer.

[15] López-Knowles E (2016). Heterogeneity in global gene expression profiles between biopsy specimens taken peri-surgically from primary ER-positive breast carcinomas. Breast Cancer Res..

[16] Gao Q (2018). Major Impact of Sampling Methodology on Gene Expression in Oestrogen Receptor–Positive Breast Cancer. JNCI Cancer Spectr..

[17] Haynes BP (2017). Molecular changes in premenopausal ooestrogen receptor-positive primary breast cancer in Vietnamese women after oophorectomy. NPJ Breast Cancer.

[18] Pujol P (1998). Changing oestrogen and progesterone receptor patterns in breast carcinoma during the menstrual cycle and menopause. Cancer.

[19] Khan SA, Gonchoroff NJ, Miller LE (1997). Expression of pS2, c-erbB-2, and cathepsin D during the menstrual cycle in human breast cancers. Ann. Surg. Oncol..

[20] Mangia A (1998). Timing of breast cancer surgery within the menstrual cycle: tumor proliferative activity, receptor status and short-term clinical outcome. J. Exp. Clin. Cancer Res..

[21] Coradini D, Veneroni S, Pellizzaro C, Daidone MG (2003). Fluctuation of intratumor biological variables as a function of menstrual timing of surgery for breast cancer in premenopausal patients. Ann. Oncol..

[22] Vasei M, Azarpira N, Talei A (2006). Status of oestrogen and progesterone receptors in various phases of the menstrual cycle in breast cancer. Arch. Iran. Med.

[23] Atalay C, Kanliöz M, Altinok M (2002). Menstrual cycle and hormone receptor status in breast cancer patients. Neoplasma..

[24] Pujol P (2001). A prospective prognostic study of the hormonal milieu at the time of surgery in premenopausal breast carcinoma. Cancer.

[25] Saad Z (1998). Expression of genes that contribute to proliferative and metastatic ability in breast cancer resected during various menstrual phases. Lancet.

[26] Mohammed H (2015). Progesterone receptor modulates ERα action in breast cancer. Nature.

[27] Singhal H (2016). Genomic agonism and phenotypic antagonism between oestrogen and progesterone receptors in breast cancer. Sci. Adv..

[28] Hissom JR, Moore MR (1987). Progestin effects on growth in the human breast cancer cell line T-47D — possible therapeutic implications. Biochem. Biophys. Res. Commun..

[29] Liang Y, Besch-Williford C, Brekken RA, Hyder SM (2007). Progestin-dependent progression of human breast tumor xenografts: a novel model for evaluating antitumor therapeutics. Cancer Res..

[30] Giulianelli S (2012). Oestrogen receptor alpha mediates progestin-induced mammary tumor growth by interacting with progesterone receptors at the *Cyclin D1*/*MYC* promoters. Cancer Res..

[31] Kabos P (2012). Patient-derived luminal breast cancer xenografts retain hormone receptor heterogeneity and help define unique oestrogen-dependent gene signatures. Breast Cancer Res. Treat..

[32] Wanifuchi-Endo Y (2019). Effects of serum oestradiol and progesterone on oestrogen-regulated gene expression in breast cancers of premenopausal patients. Jpn J. Clin. Oncol..

[33] Chapman SC, Woodruff TK (2001). Modulation of activin signal transduction by inhibin B and inhibin-binding protein (INhBP). Mol. Endocrinol..

[34] Seidah NG, Arbatti NJ, Rochemont J, Sheth AR, Chrétien M (1984). Complete amino acid sequence of human seminal plasma beta-inhibin. Prediction of post Gln-Arg cleavage as a maturation site. FEBS Lett..

[35] Tanos T (2013). Progesterone/RANKL is a major regulatory axis in the human breast. Sci. Transl. Med..

[36] Gonzalez-Suarez E (2010). RANK ligand mediates progestin-induced mammary epithelial proliferation and carcinogenesis. Nature.

[37] Hu H (2014). RANKL expression in normal and malignant breast tissue responds to progesterone and is up-regulated during the luteal phase. Breast Cancer Res. Treat..

[38] Wirapati P (2008). Meta-analysis of gene expression profiles in breast cancer: toward a unified understanding of breast cancer subtyping and prognosis signatures. Breast Cancer Res..

[39] Simigdala N (2016). Cholesterol biosynthesis pathway as a novel mechanism of resistance to oestrogen deprivation in oestrogen receptor-positive breast cancer. Breast Cancer Res..

[40] Lee JS (2006). Comparison of methods to measure low serum oestradiol levels in postmenopausal women. J. Clin. Endocrinol. Metab..

[41] Dowsett M (2005). Biomarker changes during neoadjuvant anastrozole, tamoxifen, or the combination: influence of hormonal status and HER-2 in breast cancer–a study from the IMPACT trialists. J. Clin. Oncol..

[42] Zabaglo L (2010). Comparative validation of the SP6 antibody to Ki67 in breast cancer. J. Clin. Pathol..

[43] Richer JK (2002). Differential gene regulation by the two progesterone receptor isoforms in human breast cancer cells. J. Biol. Chem..

[44] Robker RL (2000). Progesterone-regulated genes in the ovulation process: ADAMTS-1 and cathepsin L proteases. Proc. Natl Acad. Sci. USA.

[45] Wood CE, Register TC, Cline JM (2009). Transcriptional profiles of progestogen effects in the postmenopausal breast. Breast Cancer Res. Treat..

[46] Purmonen S, Manninen T, Pennanen P, Ylikomi T (2008). Progestins regulate genes that can elicit both proliferative and antiproliferative effects in breast cancer cells. Oncol. Rep..

[47] Leo JC (2005). Gene regulation profile reveals consistent anticancer properties of progesterone in hormone-independent breast cancer cells transfected with progesterone receptor. Int. J. Cancer.

[48] Mrusek S, Classen-Linke I, Vloet A, Beier HM, Krusche CA (2005). Oestradiol and medroxyprogesterone acetate regulated genes in T47D breast cancer cells. Mol. Cell. Endocrinol..

[49] Geiss GK (2008). Direct multiplexed measurement of gene expression with color-coded probe pairs. Nat. Biotechnol..

[50] Haynes, B. P. et al. Metadata supporting data files in the published article: Menstrual cycle associated changes in hormone related gene expression in oestrogen receptor positive breast cancer. figshare. Dataset. 10.6084/m9.figshare.9892211 (2019). 10.1038/s41523-019-0138-2 PMC6858333 31754627

