# Supplementary material for: Menstrual cycle associated changes in hormone-related gene expression in oestrogen receptor positive breast cancer
Source: NPJ Breast Cancer. 2019 Nov 15;5:42. doi: 10.1038/s41523-019-0138-2 (PMC6858333; doi:10.1038/s41523-019-0138-2)
Supplement: Supplementary file 2 — Supplementary information [file 41523_2019_138_MOESM2_ESM.pdf]

Supplementary Information

Menstrual cycle associated changes in hormone related gene expression in oestrogen receptor positive breast cancer

Ben P Haynes* et al

*corresponding author; Ben P. Haynes, The Ralph Lauren Centre for Breast Cancer Research, Royal Marsden Hospital, Fulham Road, London SW3 6JJ, UK. Tel. +44 20 7808 2969; Fax +44 20 3763918; E-mail: ben.haynes@icr.ac.uk

Supplementary Data 1: Nanostring raw gene expression data and window of cycle for all samples (n=130; 65 pairs)

Supplementary Data 2: Changes in gene expression between the same window and individual windows of the menstrual cycle

Supplementary Data 3: Housekeeper normalized log-transformed gene expression data for all samples (n=130; 65 pairs)

Supplementary Table 1: Patient demographics in the two independent prospective studies used

	Vietnamese study (n=20)	MenCER (n=45)
**Age in years**		
Median (range)	40 (31-49)	46 (34-49)
<35	6 (30.0%)	2 (4.4%)
35-39	4 (20.0%)	1 (2.2%)
40-44	7 (35.0%)	14 (31.1%)
>=45	3 (15.0%)	28 (62.2%)
**Lymph node status;**		
0 positive nodes	8 (40.0%)	38 (84.4%)
1-3 positive nodes	9 (45.0%)	6 (13.3%)
> 3 positive nodes	3 (15.0%)	1 (2.2%)
Not known	-	
**Tumour grade;**		
1	5 (25.0%)	15 (33.3%)
2	13 (65.0%)	23 (51.1%)
3	1 (5.0%)	7 (15.5%)
Not known	1 (5.0%)	-
**Tumour size;**		
Median (range)	2.0 (0.5-3.5)	2.4 (0.8-7.5)
**Tumour type**		
Invasive ductal	17 (85.0%)	40 (88.9%)
Lobular	1 (5.0%)	4 (8.9%)
Mixed	1 (5.0%)	1 (2.2%)
Not known	1 (5.0%)	0
**PR status**		
-ve	4 (20%)	4 (9%)
+ve	16 (80.0%)	41 (91%)
**HER2**		
-ve	15 (75.0%)	44 (97.5%)
+ve	5 (25.0%)	1 (2.2%)

Supplementary Table 2: Changes in gene expression between Window 1 vs. Window 2 or 3

Gene	Gene expression in W2 or W3 relative to W1 (=1.00)	Wilcoxon p-value	FDR
GREB1	1.80	0.0004	0.016
IGSF1	2.36	0.0009	0.018
MSMB	2.34	0.0048	0.035
PGR	1.96	0.0069	0.035
SERPINA3	1.51	0.0067	0.035
TFF1	2.21	0.0053	0.035
ATP6V1B1	1.41	0.0041	0.035
CELSR2	1.44	0.0049	0.035
UBE2C	1.33	0.0081	0.036
TOP2A	1.47	0.013	0.050
CDC20	1.28	0.014	0.052
PDZK1	1.75	0.020	0.063
TFF3	1.56	0.019	0.063
HMMR	1.38	0.023	0.066
SERPINA5	1.51	0.031	0.080
TNFSF10	0.86	0.032	0.080
RANKL (TNFSF11)	1.69	0.058	0.11
SGK3	1.19	0.047	0.11
MKI67	1.35	0.051	0.11
UBE2T	1.23	0.053	0.11
CLIC6	1.44	0.056	0.11
PDZK1P1	1.38	0.078	0.14
DCN	0.78	0.084	0.15
ERBB2	0.94	0.13	0.22
IRS1	1.11	0.18	0.27
AGR2	1.15	0.18	0.27
ACOX2	0.80	0.18	0.27
SHROOM3	1.21	0.20	0.29
STC2	1.24	0.21	0.29
RUNX1	1.10	0.30	0.40
AURKA	1.06	0.43	0.55
FKBP5	1.11	0.45	0.56
RASSF5	0.96	0.53	0.64
SLC2A3	1.16	0.57	0.67
EFHD1	1.03	0.63	0.72
NDP	1.09	0.71	0.79
MYB	1.07	0.78	0.84
ESR1	1.03	0.85	0.89
CTGF	0.97	0.99	0.99
TGFB3	0.99	0.99	0.99

AvERG	1.92	0.0005
AvProlif	1.29	0.013
AvProg	1.37	0.089

Supplementary Table 3: Changes in gene expression between Window 3 vs. Window 1 or 2

Gene	Gene expression in W3 relative to W1 or W2 (=1.00)	Wilcoxon p-value	FDR
FKBP5	1.49	0.0057	0.23
ATP6V1B1	0.68	0.016	0.32
SERPINA3	1.32	0.033	0.44
PDZK1	0.69	0.053	0.53
RANKL (TNFSF11)	1.44	0.10	0.65
RUNX1	1.03	0.12	0.65
EFHD1	1.09	0.12	0.65
MYB	0.87	0.13	0.65
TFF1	0.79	0.19	0.73
ACOX2	0.85	0.19	0.73
UBE2T	1.10	0.20	0.73
IGSF1	0.68	0.22	0.73
PDZK1P1	1.18	0.27	0.83
TGFB3	1.08	0.30	0.83
NDP	0.86	0.31	0.83
PGR	0.99	0.33	0.83
TFF3	0.89	0.39	0.86
CELSR2	0.94	0.41	0.86
MSMB	1.36	0.43	0.86
DCN	0.90	0.45	0.86
ERBB2	1.02	0.45	0.86
RASSF5	1.14	0.52	0.88
AURKA	0.95	0.52	0.88
TNFSF10	0.98	0.53	0.88
CTGF	1.12	0.59	0.94
SERPINA5	0.96	0.70	0.98
TOP2A	1.08	0.70	0.98
GREB1	0.97	0.72	0.98
CLIC6	0.95	0.74	0.98
STC2	0.92	0.74	0.98
AGR2	1.02	0.76	0.98
IRS1	0.97	0.78	0.98
SLC2A3	1.09	0.82	0.99
UBE2C	1.02	0.86	0.99
ESR1	1.00	0.89	0.99
CDC20	1.00	0.92	0.99
SGK3	1.01	0.93	0.99
SHROOM3	0.95	0.95	0.99
HMMR	1.06	0.98	0.99
MKI67	1.01	0.99	0.99

AvERG	0.85	0.13
AvProlif	1.01	0.98
AvProg	1.46	0.026

Supplementary Table 4: Changes in gene expression of housekeeping genes between different windows of the menstrual cycle

Gene	Gene expression relative to first window (=1.00)	Wilcoxon p-value	FDR
**W1 vs. W2 (n=14)**			
ACTB	0.97	0.50	0.61
MRPL19	0.96	0.25	0.42
PSMC4	0.92	0.01	0.11
SF3A1	1.01	0.50	0.61
TBP	1.14	0.02	0.11
**W1 vs. W3 (n=10)**			
ACTB	1.01	0.004	0.160
MRPL19	1.01	0.85	0.890
PSMC4	1.00	0.32	0.540
SF3A1	0.96	0.19	0.400
TBP	1.01	0.02	0.160
**W2 vs. W3 (n=20)**			
ACTB	1.01	0.76	0.91
MRPL19	1.01	0.55	0.90
PSMC4	1.00	0.87	0.93
SF3A1	0.96	0.21	0.72
TBP	1.01	0.57	0.90
**W1 vs W2 or W3 (n=28)**			
ACTB	1.07	0.06	0.13
MRPL19	1.01	0.91	0.95
PSMC4	0.98	0.52	0.66
SF3A1	0.97	0.35	0.49
TBP	0.96	0.65	0.75
**W1 or W2 vs. W3 (n=32)**			
ACTB	1.06	0.08	0.65
MRPL19	1.02	0.52	0.82
PSMC4	1.01	0.53	0.82
SF3A1	0.97	0.53	0.82
TBP	0.94	0.22	0.71
**Same window (n=15)**			
ACTB	1.13	0.11	0.28
MRPL19	1.04	0.18	0.38
PSMC4	0.93	0.04	0.18
SF3A1	1.05	0.06	0.20
TBP	0.94	0.19	0.39

Supplementary Figure 1

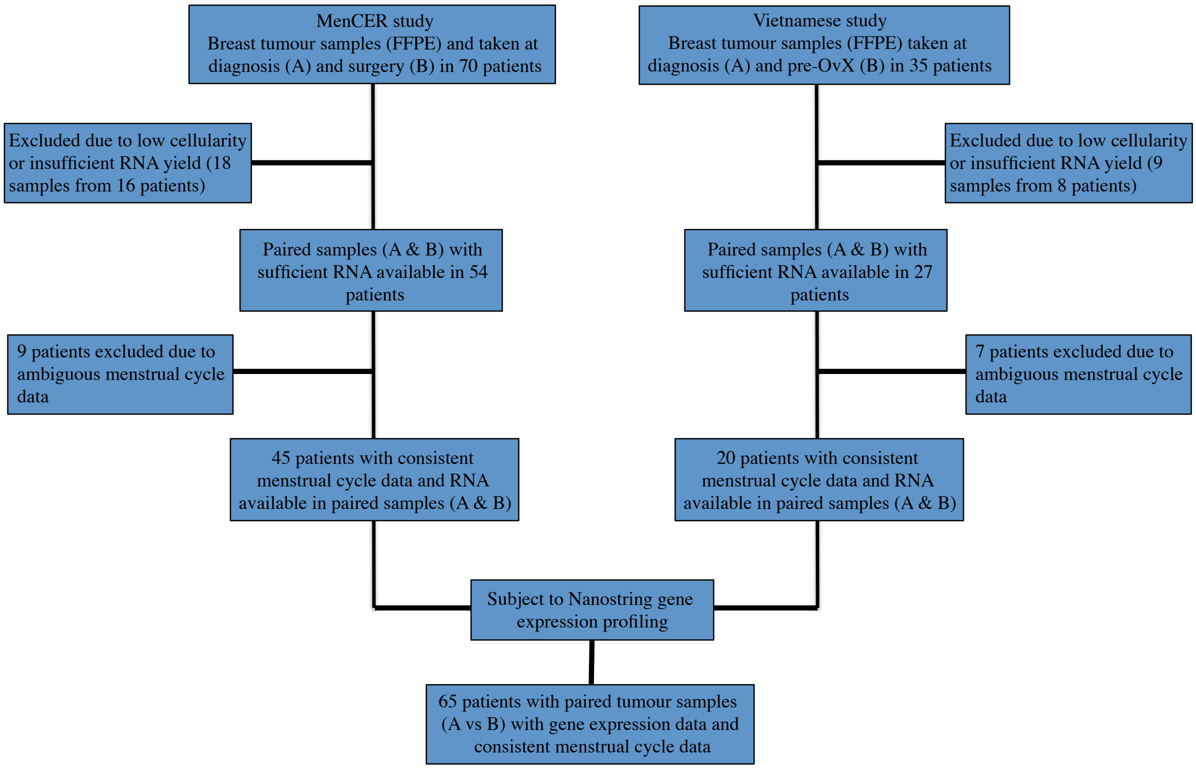

Supplementary Figure 1; Consort diagram showing the derivation of samples for gene expression studies.

Supplementary Figure 2

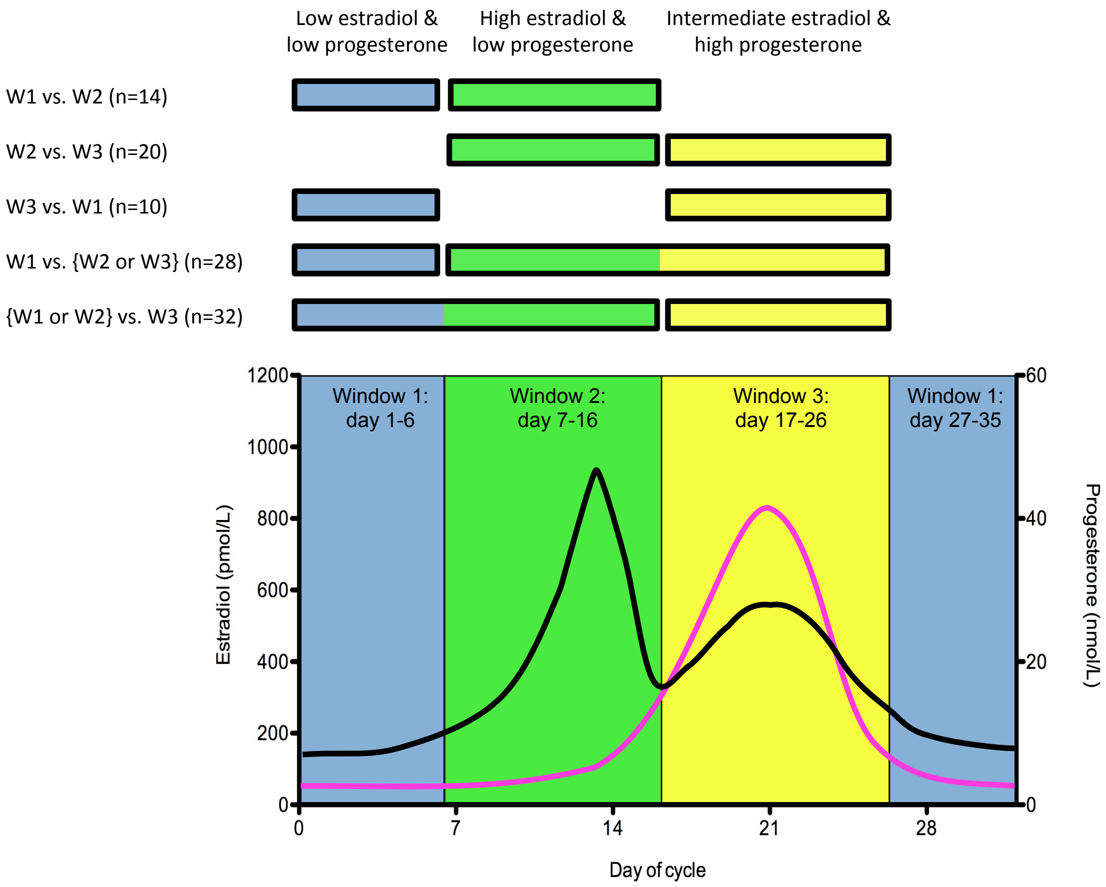

Supplementary Figure 2: Pre-defined menstrual cycle windows and number of window comparisons available.

Supplementary Figure 3

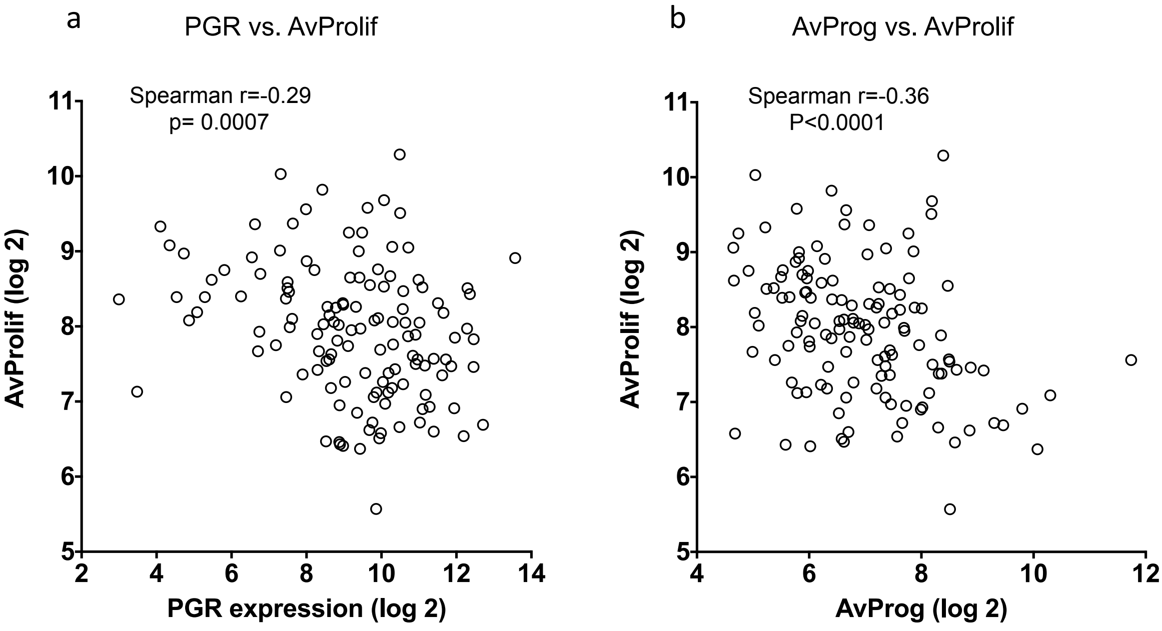

Supplementary Figure 3: Relationship between proliferation (AvProlif) and progesterone receptor expression (a; PGR) and progesterone receptor signaling (b; AvProg) and in all samples (n=130).

Supplementary Figure 4

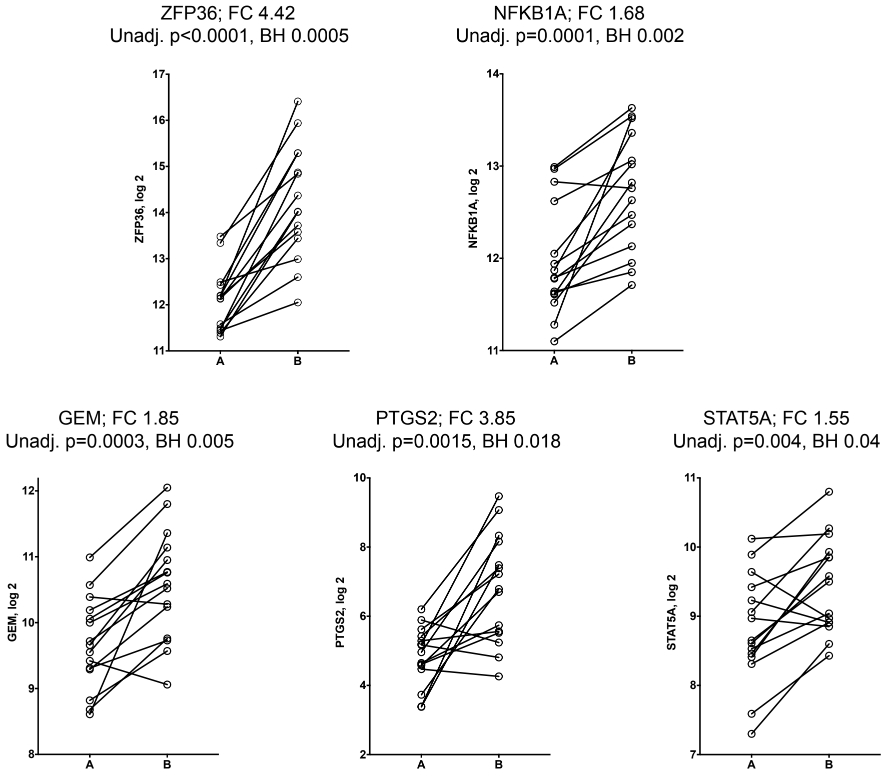

Supplementary Figure 4: Genes showing significant changes in comparisons made between samples taken in the same window.

Supplementary Figure 5

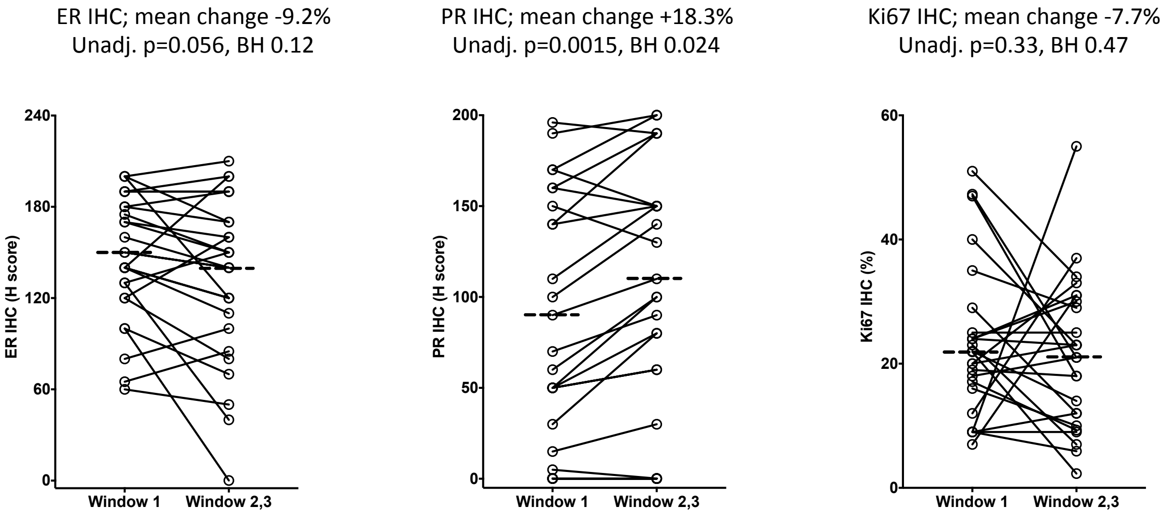

Supplementary Figure 5: Changes in protein levels of ER, PgR and Ki67 between Window 1 vs. Windows 2 or 3 (n=28). Dotted lines indicate median level of window.

Supplementary Figure 6

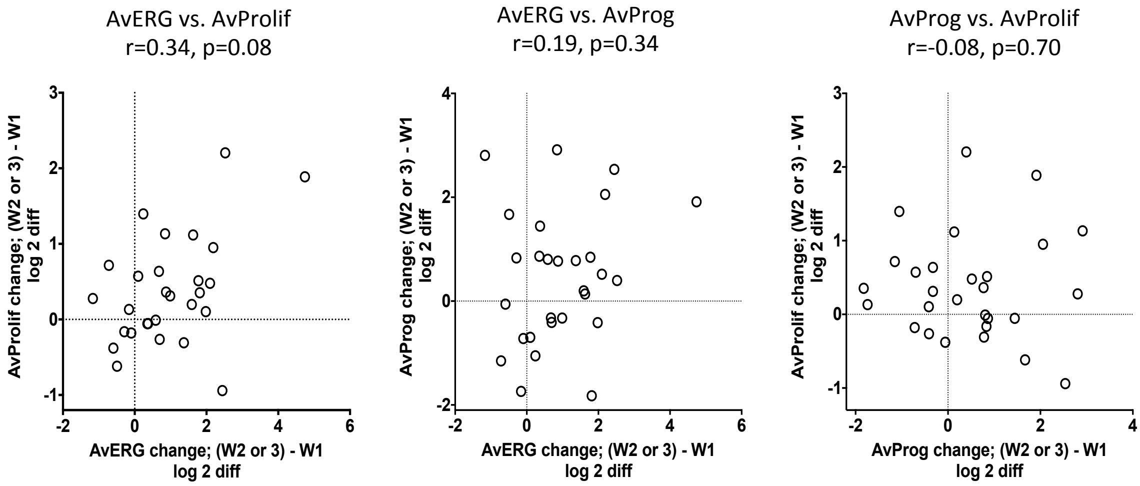

Supplementary Figure 6: Correlation of changes in AvERG, AvProlif and AvProg between Window 1 vs. Window 2 or 3 (n=28).

Supplementary Figure 7

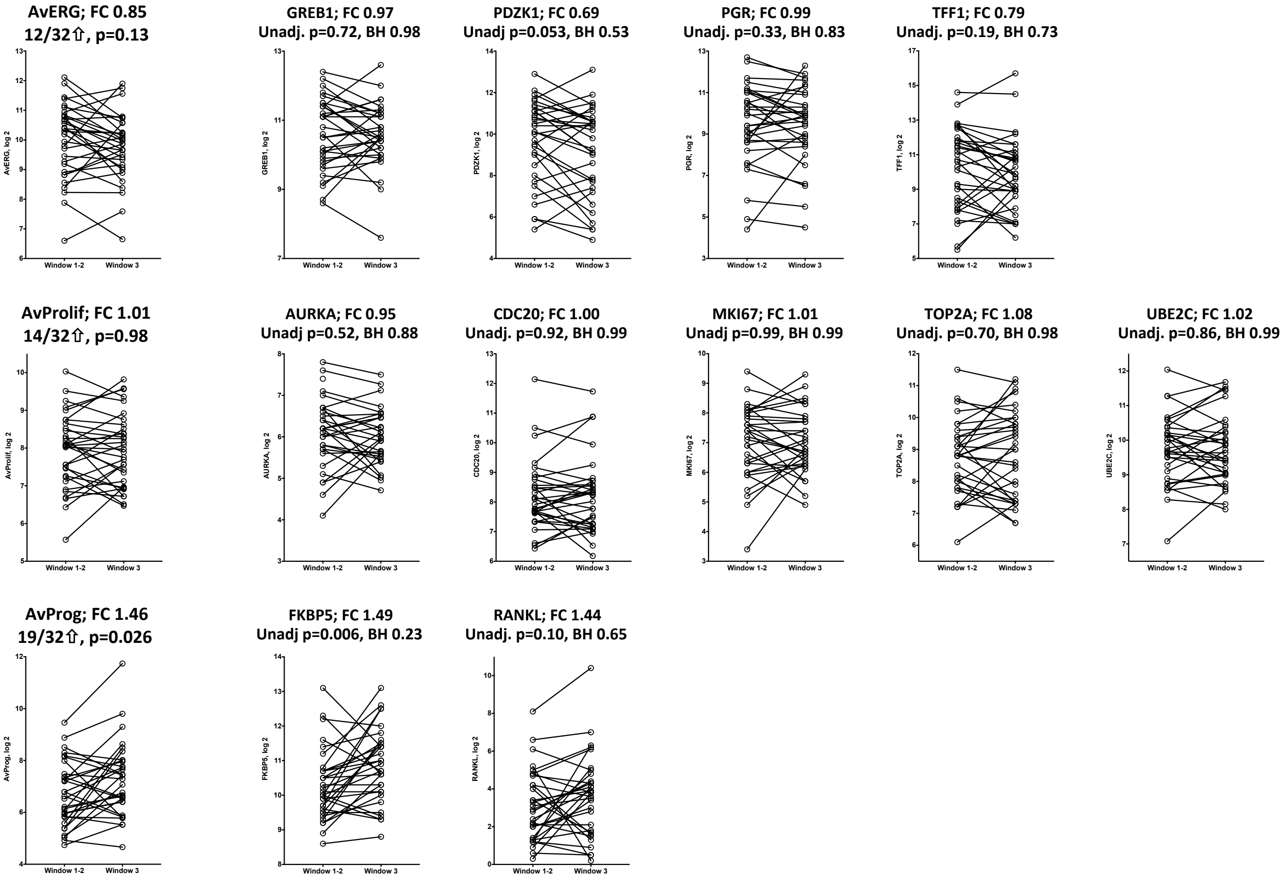

Supplementary Figure 7: Changes in AvERG, AvProlif and AvProg and in expression of their individual component ERGs, PAGs and PRGs between Window 3 vs. Window 1 or 2 (n=32)

Supplementary Figure 8

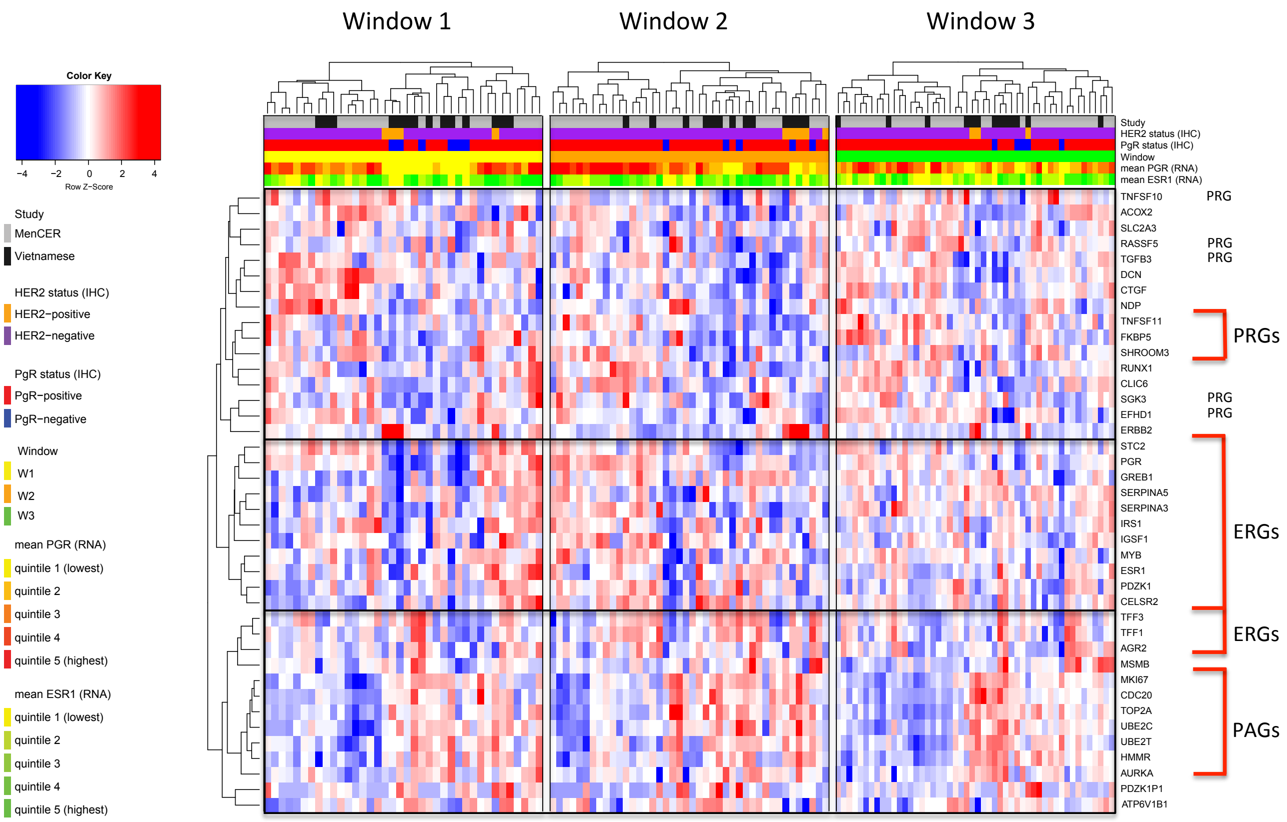

Supplementary Figure 8: Hierarchical clustering of the gene expression data from the combined sample set of 130 samples (65 pairs) according to the window of the menstrual cycle the sample was taken in (values z-scored across all three windows)
